# Supplementary material for: Synthesis of Calamitic Fluorinated Mesogens with Complex Crystallization Behavior
Source: Molecules. 2023 Dec 8;28(24):8002. doi: 10.3390/molecules28248002 (PMC10745429; doi:10.3390/molecules28248002)
Supplement: Supplementary file 1 [file molecules-28-08002-s001.zip › molecules-2730813-supplementary.pdf]

Supplementary Information.

Synthesis of Calamitic Fluorinated Mesogens with Complex Crystallization Behavior

Denis Anokhin [1,2,*], Alina Maryasevskaya [1], Ainur Abukaev [1], Umut Ugur Ozkose [3,4], Alexander Buglakov [1,5], Dimitri A. Ivanov [1,2,6] and Bruno Améduri [1,3,*]

1 Faculty of Chemistry, Lomonosov Moscow State University, GSP-1, 1-3 Leninskiye Gory, 119991 Moscow, Russia; ainurabukaev@gmail.com (A.A.); buglakov@polly.phys.msu.ru (A.B.); dimitri.ivanov@uha.fr (D.A.I.)
2 Scientific Center for Genetics and Life Sciences, Sirius University of Science and Technology, 1 Olympic Ave., 354340 Sochi, Russia
3 Institut Charles Gerhardt, CNRS, University of Montpellier, Ecole Nationale Supérieure de Chimie de Montpellier, 34000 Montpellier, France; umut-ugur.ozkose@umontpellier.fr
4 Department of Chemistry, Faculty of Science and Letters, Piri Reis University, Tuzla, 34940 Istanbul, Turkey
5 A. N. Nesmeyanov Institute of Organoelement Compounds RAS, Vavilova ul., 28, 119334 Moscow, Russia
6 Institut de Sciences des Matériaux de Mulhouse-IS2M, CNRS UMR 7361, Jean Starcky 15, 68057 Mulhouse, France
* Correspondence: deniano@yahoo.com (D.A.); bruno.ameduri@enscm.fr (B.A.); Tel.: +33-(0)4-48-79-2020 (B.A.)

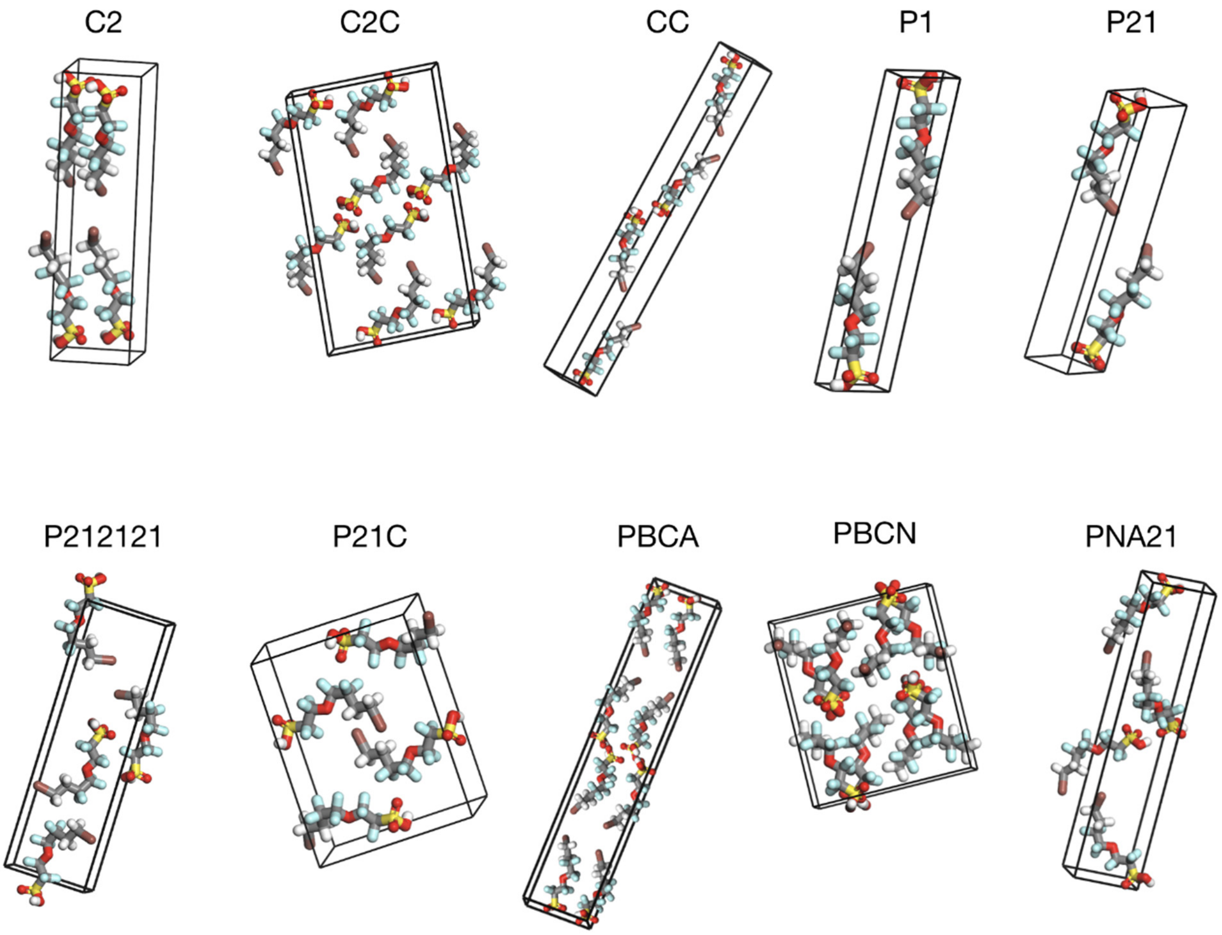

Figure S1: Possible symmetries of unit cell of $ICH_2CH_2CF_2CF_2OCF_2CF_2SO_3H$ used in simulation.

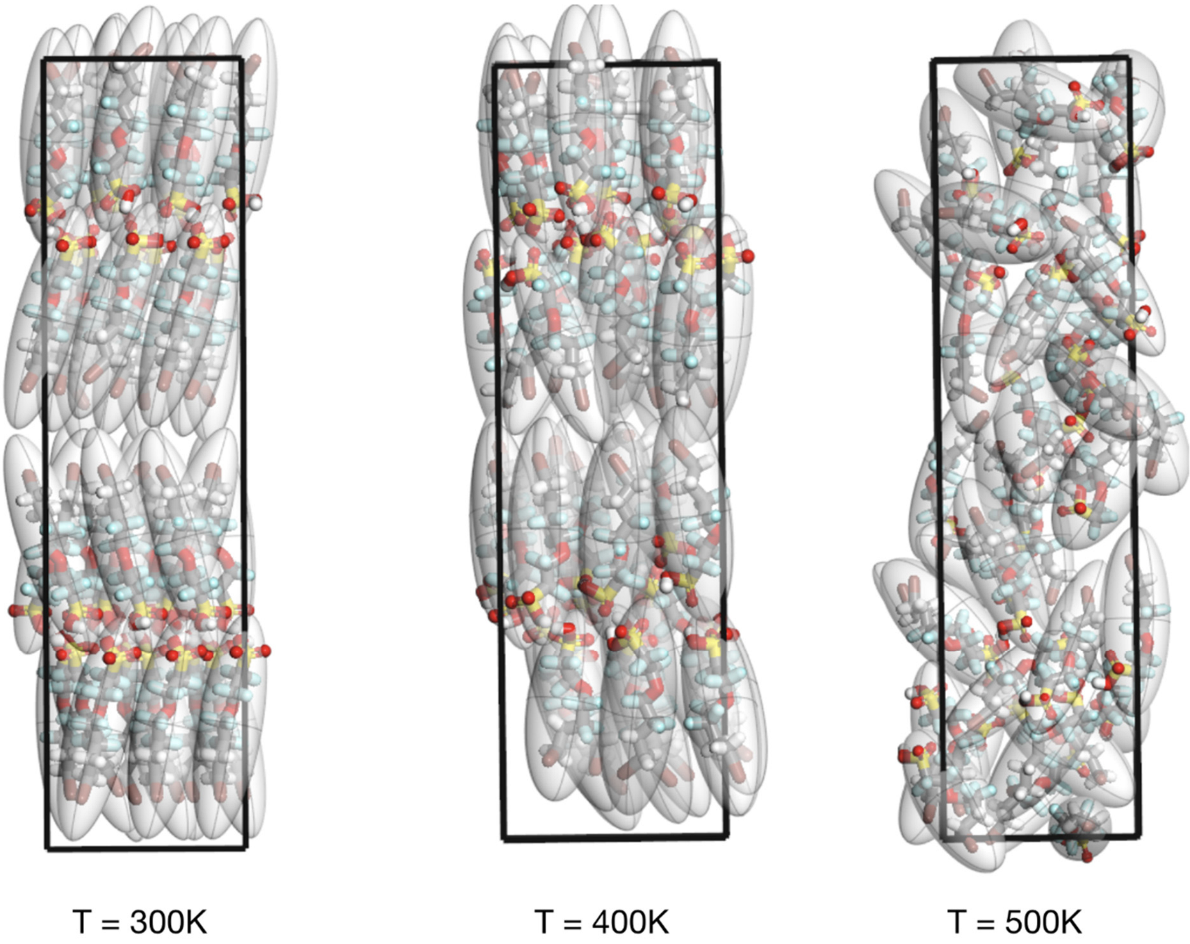

Figure S2: Model of P1 unit cell of $ICH_2CH_2CF_2CF_2OCF_2CF_2SO_3H$ at different temperatures.

Table S1: Experimental and theoretical peak positions for $ICH_2CH_2CF_2CF_2OCF_2CF_2SO_3H$ at room temperature.

h	*k*	*l*	d_{exp}	d_{calc}
1	0	0	27.54	27.50
2	0	0	13.78	13.80
3	0	0	9.17	9.18
0	1	0	5.21	5.16
1	1	0	5.04	5.08
0	0	1	4.96	4.97
1	0	1	4.87	4.89
2	0	-1	4.75	4.67
6	0	0	4.57	4.59
3	0	1	4.39	4.37
4	-1	0	4.14	4.13
4	0	1	3.99	4.03
2	1	1	3.72	3.74
5	0	1	3.65	3.69
8	0	0	3.47	3.44
2	1	-1	3.21	3.24
4	1	1	3.39	3.38
1	-1	1	3.31	3.34
2	-1	1	3.21	3.24
3	-1	1	3.12	3.14
7	0	1	3.05	3.08
6	1	1	2.93	2.97
6	1	-1	2.67	2.70
1	2	0	2.56	2.57
2	2	0	2.51	2.54
1	1	2	2.36	2.38
4	0	2	2.32	2.34
5	0	2	2.27	2.26
6	0	2	2.18	2.18
8	2	0	2.06	2.07
8	0	2	2.02	2.01
3	2	2	1.90	1.90
4	2	2	1.86	1.86
5	2	2	1.82	1.83
6	2	2	1.78	1.79
0	3	0	1.72	1.72
0	2	-2	1.68	1.67
3	2	-2	1.64	1.64

Figure S3: structure of di(*tert*–butylcyclohexylperoxy dicarbonate

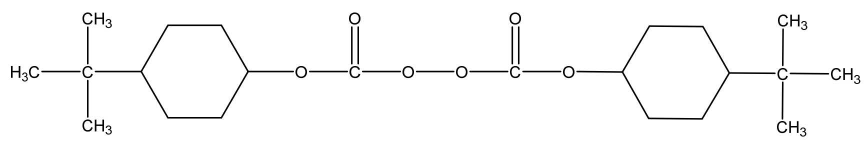

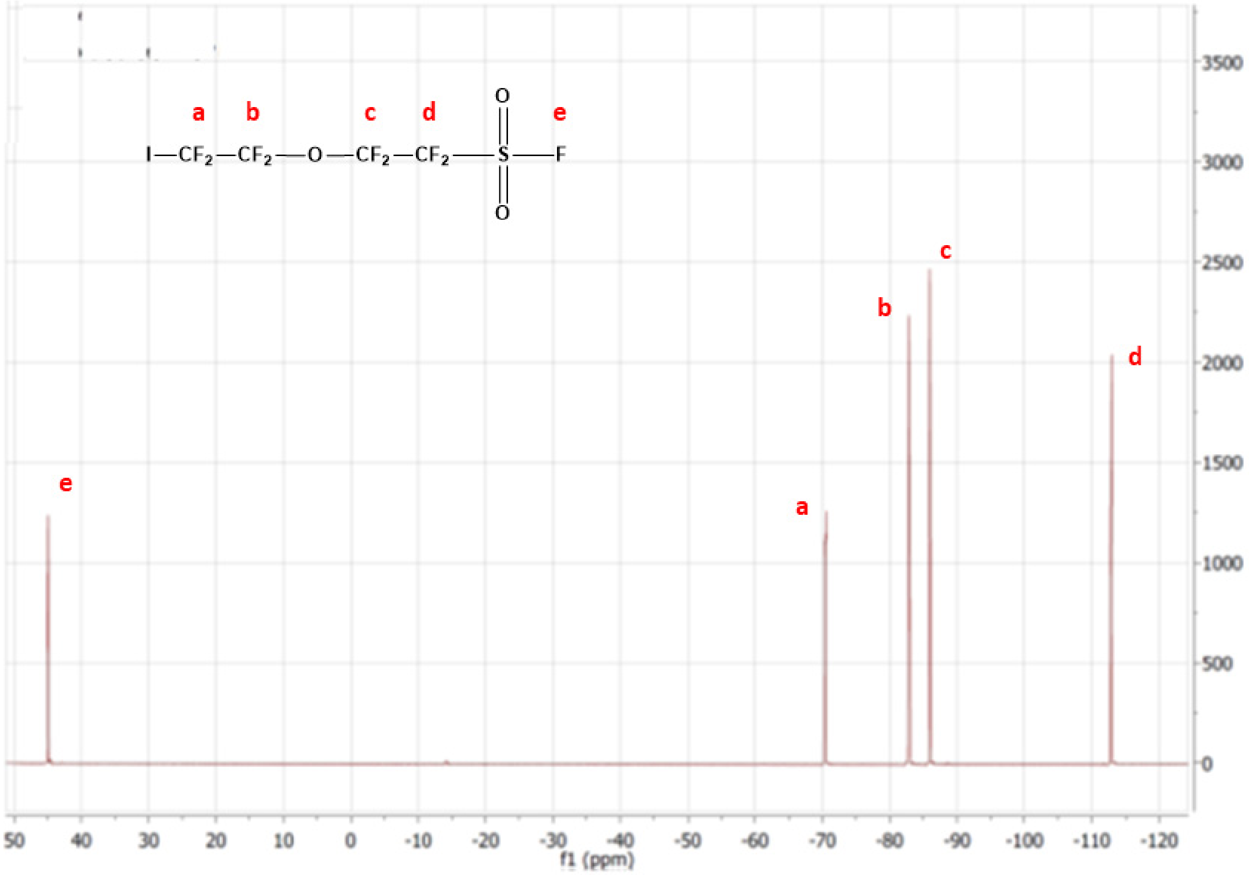

Figure S4: ^{19}F–NMR spectrum of $ICF_2CF_2OCF_2CF_2SO_2F$ recorded in d_6–acetone.

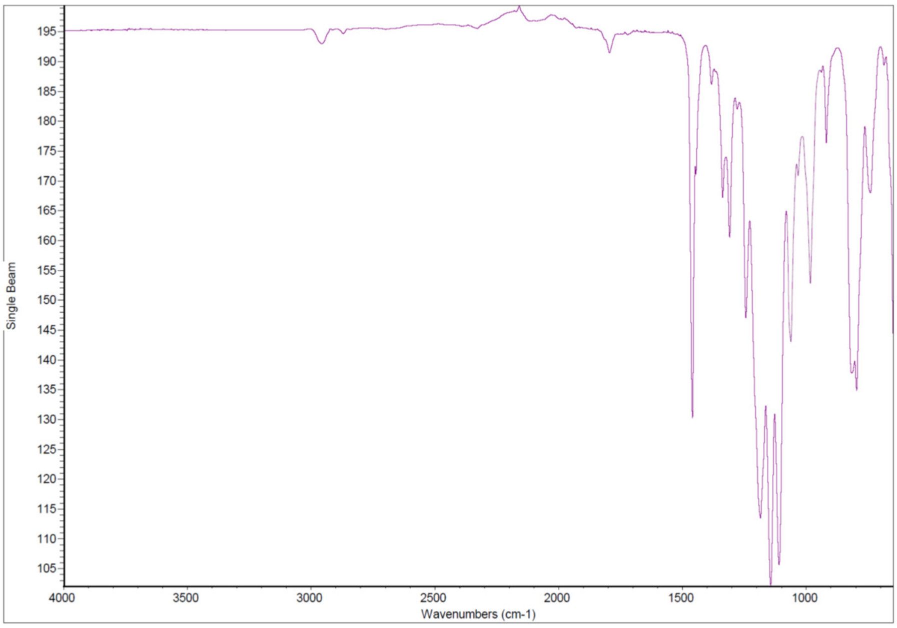

Figure S5: FT-IR spectrum of $ICH_2CH_2CF_2CF_2OCF_2CF_2SO_2F$

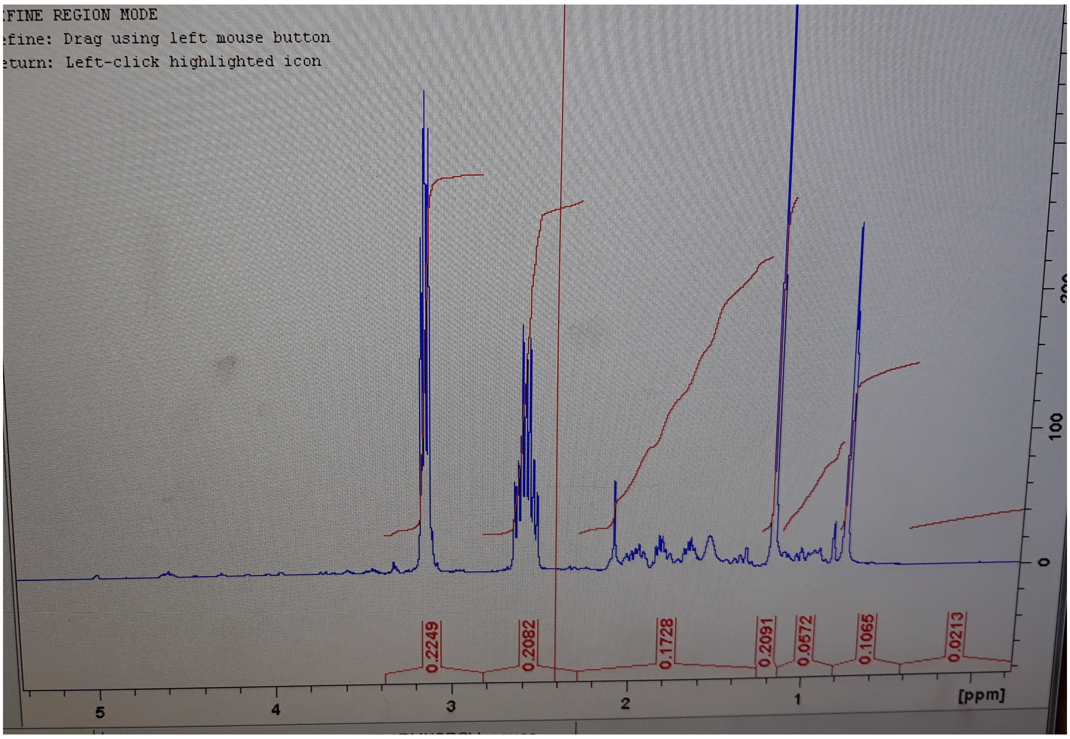

Figure S6: . ^{1}H–NMR spectrum of $ICH_2CH_2CF_2CF_2OCF_2CF_2SO_2F$ recorded in d_6–acetone.

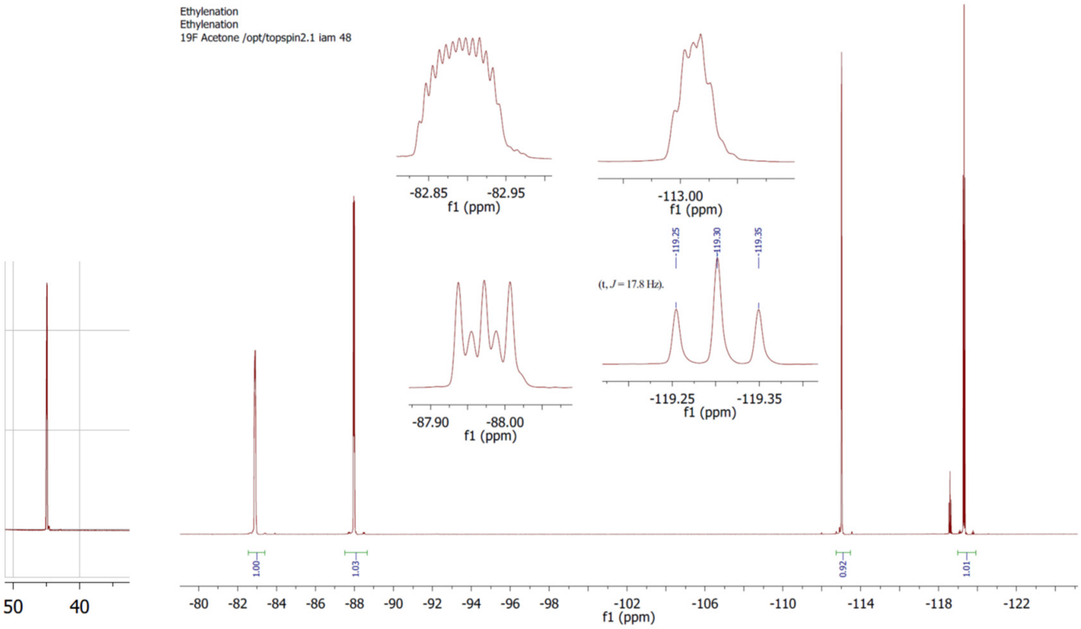

Figure S7 ^{19}F NMR spectrum of $I\text{-}CH_2CH_2\text{-}C_2F_4\text{-}O\text{-}C_2F_4\text{-}SO_2F$ in d6-acetone

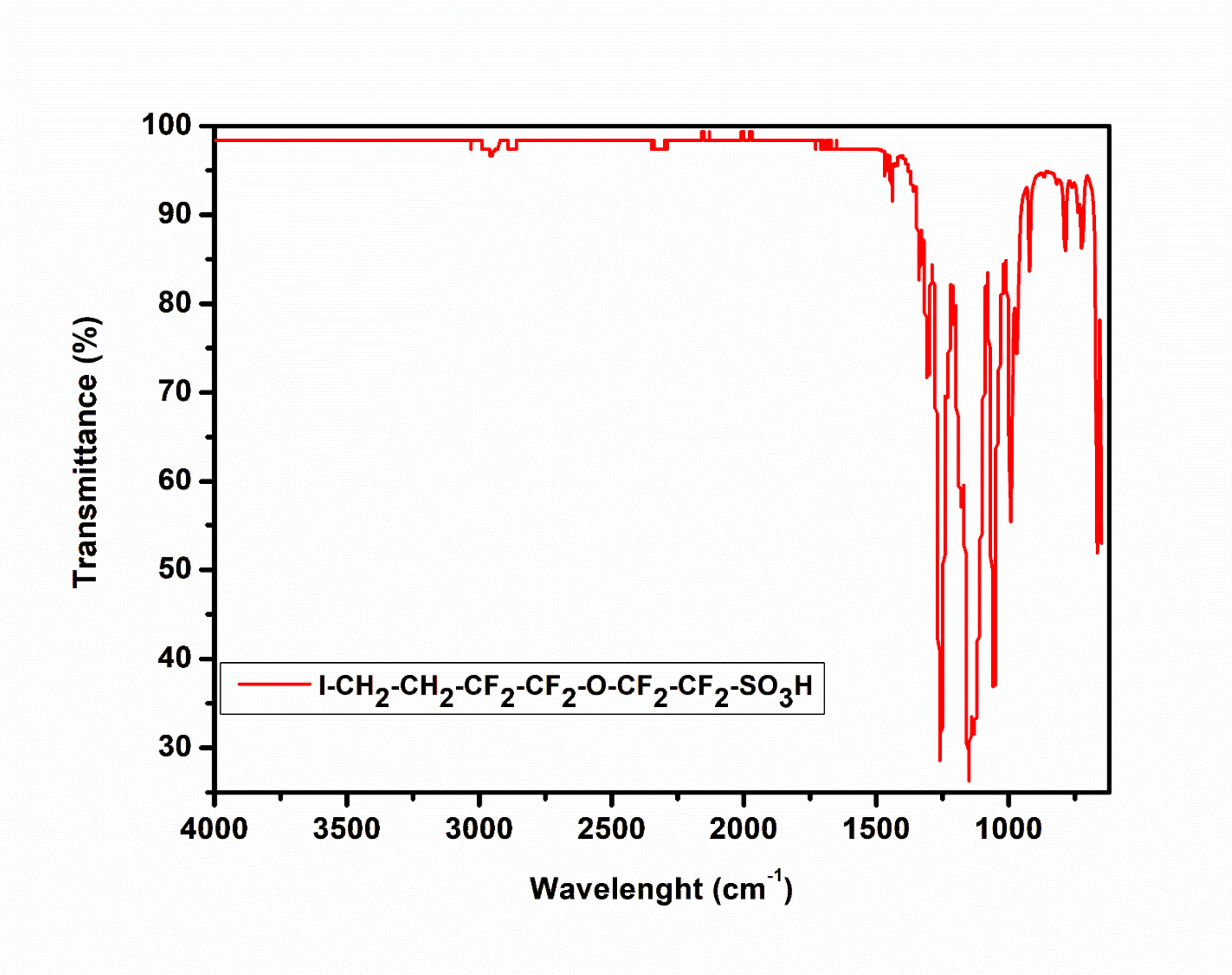

Figure S8: FT-IR spectrum of $ICH_2CH_2CF_2CF_2OCF_2CF_2SO_3H$.

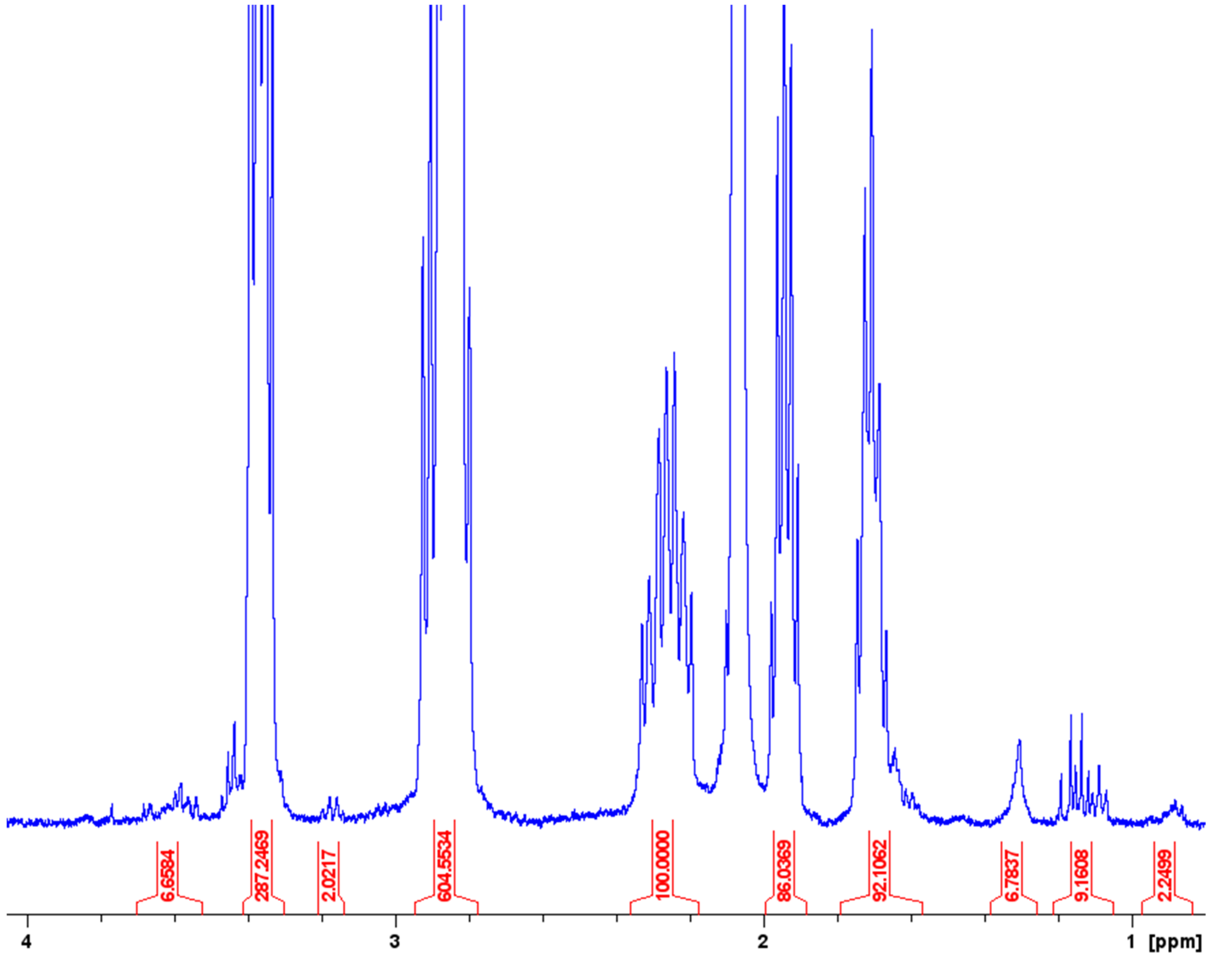

Figure S9: ^{1}H–NMR spectrum of $ICH_2CH_2CF_2CF_2OCF_2CF_2SO_3H$ recorded in d_6–acetone.

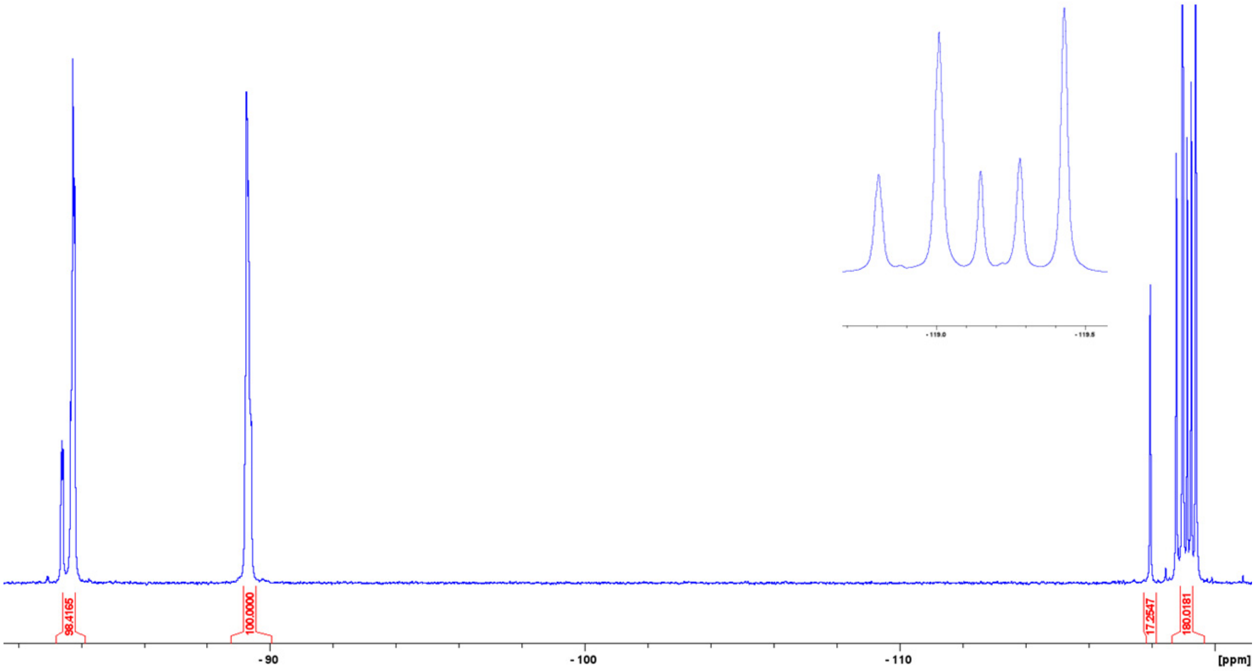

Figure S10: ^{19}F–NMR spectrum of $ICH_2CH_2CF_2CF_2OCF_2CF_2SO_3H$ recorded in d_6–acetone.
